# Impact of Neutral Sphingomyelinase Inhibition on Small Extracellular Vesicle Production by Mural Granulosa Cells and In Vitro Folliculogenesis in Mice

**DOI:** 10.1002/mrd.70063

**Published:** 2025-10-16

**Authors:** Kodai Matsushita, Yuta Matsuno, Kazuma Kita, Ayaka Ichikawa, Natsumi Maruyama, Wataru Fujii, Tsutomu Endo, Koji Sugiura

**Affiliations:** ^1^ Department of Animal Resource Sciences, Graduate School of Agricultural and Life Sciences The University of Tokyo Tokyo Japan; ^2^ Department of Veterinary Medical Sciences, Graduate School of Agricultural and Life Sciences The University of Tokyo Tokyo Japan

**Keywords:** extracellular vesicles, granulosa cells, mice, ovarian follicles

## Abstract

Small extracellular vesicles (sEVs) function as critical regulators of ovarian follicular development. Although several pathways, including one involving neutral sphingomyelinase (nSMase), contribute to sEV production, the specific pathway active in ovarian follicles has not been clearly identified. In this study, we investigated GW4869, a specific inhibitor of nSMase activity, to determine its impact on sEV production by mouse mural granulosa cells (MGCs), the primary source of follicular sEVs. We also examined how nSMase inhibition affects the in vitro growth of oocyte‒granulosa cell complexes (OGCs) derived from secondary follicles. Transcripts encoding nSMases (*Smpd2* and *Smpd4*) were detected in MGCs, and GW4869 treatment significantly reduced sEV production in MGC monolayer cultures. Control OGCs developed into antral follicle‐like structures, with the antrum‐like structure separating granulosa cells into cumulus‐like and MGC‐like cells. However, GW4869 treatment impaired OGC development. MGC‐like cells from GW4869‐treated OGCs exhibited significantly lower *Cyp19a1* levels, whereas adding MGC‐derived sEVs promoted *Cyp19a1* expression. These results suggest that nSMase activity, likely involving *Smpd2* and *Smpd4*, is required for sEV production by MGCs and that follicular sEVs may regulate *Cyp19a1* expression in MGCs.

## 1 Introduction

During follicular development in mammals, the antral cavity partitions the granulosa cells of preantral follicles into two distinct subpopulations: cumulus cells and mural granulosa cells (MGCs). Cumulus cells surround the oocyte and play a critical role in supporting its development, whereas MGCs line the follicular wall and are primarily involved in the follicle's endocrine functions. Proper differentiation and functional specialization of these cell types are crucial for normal follicular development and female fertility.

Follicular development is regulated by a diverse array of intra‐ and extra‐follicular factors (Orisaka et al. 2021; Sugiura et al. 2023). For example, growth factors secreted by oocytes induce preantral granulosa cells (PAGCs) located near the oocytes to differentiate into cumulus cells, whereas differentiation into MGCs largely depends on stimulation by an extrafollicular factor, follicle‐stimulating hormone (FSH) (Diaz et al. 2007a, 2007b). Estrogens, produced mainly by MGCs, also play critical roles in the differentiation of both cumulus cells and MGCs (Emori et al. 2022, 2013; Ito et al. 2022; Sugiura et al. 2010). In addition to these, numerous other factors contribute to follicular development, but a comprehensive understanding of the underlying mechanisms remains incomplete.

Extracellular vesicles (EVs), including exosomes (50–150 nm) and microvesicles (100–1000 nm), are cell‐derived membranous structures that contain bioactive components and mediate intercellular communication. Exosomes and microvesicles differ in their biogenesis pathways: exosomes are generated through the endocytic pathway, whereas microvesicles are formed by budding from the plasma membrane. Because the specific pathways of EV formation are difficult to determine, these vesicles are often categorized by size rather than biogenesis. EVs ranging in size from 30 to 200 nm are commonly referred to as small EVs (sEVs).

Following the initial report in mares (da Silveira et al. 2012), many studies have demonstrated the presence of sEVs in the follicular fluid of various mammalian species, including cattle (Sohel et al. 2013), pigs (Matsuno et al. 2019), and humans (Diez‐Fraile et al. 2014; Santonocito et al. 2014). Follicular sEVs promote granulosa cell proliferation in cattle (Hung et al. 2017) and pigs (Yuan et al. 2021), affect steroidogenesis in porcine granulosa cells (Yuan et al. 2021), and promote or facilitate cumulus expansion by bovine and porcine cumulus‒oocyte complexes (COCs) (Hung et al. 2015; Matsuno et al. 2017). In addition, follicular sEVs have been reported to enhance developmental competence (da Silveira et al. 2017) and protect oocytes from heat stress in cattle (Rodrigues et al. 2019). Furthermore, follicular sEVs are implicated in ovarian pathogenesis, as specific microRNAs contained in follicular sEVs of polycystic ovary syndrome (PCOS) patients have been shown to inhibit proliferation (Yuan et al. 2021), provoke apoptosis (Zhao et al. 2022), and induce cellular senescence (Yuan et al. 2021) in primary granulosa cells and/or granulosa tumor cell lines. Therefore, it is evident that follicular sEVs are critical regulators of follicular development in mammals (de Ávila and da Silveira 2019; Qamar et al. 2020). However, further research is needed to fully elucidate the complex mechanisms by which sEVs exert their diverse functions within the follicle.

The mechanisms underlying sEV biogenesis vary depending on cell type and physiological state (van Niel et al. 2018). Fundamentally, exosomes, a major component of sEVs, are intraluminal vesicles (ILVs) formed through the inward budding of the endosomal membrane during the maturation of multivesicular bodies (MVBs). These ILVs carry membrane cargo and molecules from the surrounding cytoplasm and are subsequently secreted when MVBs fuse with the plasma membrane. While the endosomal sorting complex required for transport (ESCRT) plays a critical role in controlling cargo selection and ILV budding into MVBs, exosome biogenesis can also occur in the absence of ESCRTs. The ESCRT‐independent pathway for exosome biogenesis requires the generation of ceramide by neutral sphingomyelinase (nSMase) (Trajkovic et al. 2008). Indeed, exosome production is suppressed by the nSMase inhibitor GW4869 in several cell types, including mouse macrophages (Essandoh et al. 2015), Lewis lung carcinoma cells (Hu et al. 2018), and a human myeloma cell line (Cheng et al. 2018). Additional ESCRT‐independent pathways involving tetraspanins have also been described (van Niel et al. 2011). Therefore, various mechanisms may act in parallel or succession in exosome biogenesis. Although follicular sEVs are produced mainly by MGCs (Matsuno et al. 2019), the mechanism underlying sEV production by MGCs remains poorly understood.

To gain insight into this mechanism, we assessed the effects of nSMase inhibition (i.e., GW4869 treatment) on sEV production by mouse MGCs. Additionally, to explore the roles of sEVs in follicular development, we examined the effects of nSMase inhibition on the in vitro growth of oocyte‒granulosa cell complexes (OGCs) derived from preantral (secondary) follicles.

## 2 Materials and Methods

### 2.1 Mice

(C57BL/6N × DBA/2) F1 mice (B6D2F1 mice) were either purchased from Sankyo Lab Services Corporation (Tokyo, Japan) or bred and maintained in the research colonies of the authors at the University of Tokyo. The Animal Care and Use Committee of the University of Tokyo approved all experiments.

### 2.2 Culture of MGCs and Isolation of sEV‐Enriched Fractions

The basic culture medium used was Minimum Essential Medium Alpha (MEMα, Thermo Fisher Scientific, Gaithersburg, MD, USA) supplemented with 2.2 mg/mL NaHCO_3_, 75 µg/mL penicillin G (Meiji Seika Pharma, Tokyo, Japan), and 50 µg/mL streptomycin sulfate (Meiji Seika Pharma).

MGCs were isolated from the ovaries of 3‐week‐old B6D2F1 female mice stimulated with pregnant mare serum gonadotropin (PMSG), as reported previously (Edure et al. 2024). Briefly, isolated MGCs were resuspended in the basic culture medium supplemented with 5% fetal bovine serum (FBS; Serana Europe GmbH, Pessin, Germany) and 3 mg/mL bovine serum albumin (BSA; Sigma‐Aldrich, St. Louis, MO, USA) and seeded at 5.0 × 10^6^ cells (for nanoparticle tracking analysis (NTA)) or 8.0 × 10^5^ cells (for other experiments) per 60 mm dish pre‐coated with E‐C‐L cell attachment matrix (Merck Millipore, Darmstadt, Germany). After 24 h of culture, the medium was replaced with 3 mL of serum‐ and BSA‐free basic culture medium, and the cells were cultured for an additional 48 h to condition the medium.

The sEV‐enriched fractions were then isolated from the MGC‐conditioned medium described above, as previously described (Matsuno et al. 2020). Briefly, after 48 h of additional culture, the conditioned medium was collected and centrifuged at 2000*g* at 4°C for 30 min. The resulting supernatant was filtered through a 0.22 µm syringe filter (Merck Millipore) and then concentrated to approximately 100 µL using a centrifugal ultrafiltration filter unit with a 100,000 MWCO (AS ONE Corporation, Osaka, Japan) to remove small molecules. Fractions enriched in sEVs were isolated from the concentrated conditioned medium using Total Exosome Isolation reagent (from cell culture media) (Thermo Fisher Scientific) according to the manufacturer's instructions. In some experiments, the medium was supplemented with up to 20 µM GW4869 (Cayman Chemical Company, Ann Arbor, MI, USA) or with its solvent, dimethyl sulfoxide (DMSO; Sigma‐Aldrich), at a final concentration of 0.2% (equivalent to the concentration in the 20 μM inhibitor group). Cell viability after 24 h of GW4869 treatment was assessed using the Cell Counting Kit‐8 (Dojindo, Kumamoto, Japan), according to the manufacturer's instructions.

### 2.3 Western Blot Analysis

Western blot analysis was conducted as reported previously (Matsuno et al. 2020), using 10 µg protein equivalent of MGC cell lysate or the entire sEV‐enriched fraction. The primary antibodies used were anti‐LAMP1 rat antibody (1:500; Cat. No. sc‐19992; Santa Cruz Biotechnology, Dallas, TX, USA), anti‐HSPA8 rat antibody (1:3000; Cat. No. GTX19136; GeneTex Inc., Irvine, CA, USA), anti‐albumin (ALB) mouse antibody (1:100; Cat. No. sc‐271605; Santa Cruz Biotechnology), and anti‐cytochrome c (CYCS) mouse antibody (1:500; Cat. No. sc‐13156; Santa Cruz Biotechnology). The secondary antibodies used were horseradish peroxidase (HRP)‐conjugated anti‐rat IgG (1:3000; Cat. No. 81‐9520; Invitrogen, Carlsbad, CA, USA), and anti‐mouse IgG antibody (1:3000; Cat. No. 115‐035‐044; Jackson ImmunoResearch, West Grove, PA, USA).

### 2.4 Nanoparticle Tracking Analysis (NTA) of the sEV‐Enriched Fractions

NTA was performed as previously reported (Matsuno et al. 2020). Briefly, sEV‐enriched fractions isolated from 3 mL of MGC‐conditioned medium were resuspended in 100 µL of PBS and analyzed using a Nanoparticle Characterization System (NanoSight LM10, Malvern Instruments, Malvern, UK) with NTA3.1 software (Build 3.1.46). Three 30‐s videos were recorded for each sample. All post‐acquisition settings were set to automatic. The particle concentrations were determined by subtracting the background values measured in PBS.

### 2.5 Transmission Electron Microscopy (TEM)

Transmission electron microscope was used to examine sEV‐enriched fractions by the negative stain method, as previously described (Matsuno et al. 2017). Briefly, the sEV‐enriched fraction was resuspended in NaHCa buffer (30 mM HEPES, 100 mM NaCl, 2 mM CaCl₂, pH 7.4) and applied to a 200‐mesh copper microgrid coated with a formvar support film, which had been pretreated using soft plasma etching equipment (SEDE‐AF, Meiwafosis Co. Ltd., Tokyo, Japan). The microgrid was then stained with 1% uranyl acetate solution for 10 min, followed by staining with lead acetate solution for 10 min. Finally, the specimens were examined using a transmission electron microscope (JEM‐1400Plus; JEOL, Tokyo, Japan).

### 2.6 Total RNA Extraction, cDNA Synthesis, and Reverse Transcription‒Polymerase Chain Reaction (RT‐PCR)

Each organ and MGCs were recovered from 3‐week‐old B6D2F1 female mice. Total RNA was extracted using TRIzol Reagent (Invitrogen) and reverse‐transcribed using SuperScript Ⅳ Reverse Transcriptase (Invitrogen) in accordance with the manufacturer's instructions. PCR was performed using KOD FX Neo polymerase (TOYOBO, Osaka, Japan). Amplification was carried out with an initial denaturation at 95°C for 5 min, followed by 32 cycles of denaturation at 95°C for 30 s, annealing at 56°C for 30 s, and elongation at 72°C for 90 s. A final elongation step was performed at 72°C for 10 min. For *Actb*, the annealing temperature was 57°C, and 28 cycles were employed. The PCR products were separated by agarose gel electrophoresis, and the DNA bands were visualized using ethidium bromide staining. The PCR primers used in this study are listed in Table 1.

**Table 1:** Primer sets used for PCR.

Gene	Refseq Acc. no.	Forward primer sequence (5′−3′)	Reverse primer sequence (5′−3′)	Product size (bp)
*Actb*	NM_007393	GGCGGACTGTTACTGAGCTG	CCAGGGAGACCAAAGCCTTC	597
*Smpd2*	NM_009213	CAATCTCAACTGCTGGGACA	CTGCATTCTTGGATGTGTGG	476
*Smpd3*	NM_021491	TGCAGGAGGTGTTTGACAAG	TGCTGCTCCAGTTTGTCATC	490
*Smpd4*	NM_001403422	GGAGTCCTTCACGCCTACTG	CACGGTTCAGAAAGCTCACA	404

### 2.7 Reverse Transcription‒Quantitative PCR (RT‐qPCR)

Cumulus cells and MGCs were collected from 3‐week‐old B6D2F1 female mice with or without PMSG stimulation. PAGCs were isolated from 12‐day‐old mice by collagenase treatment, as reported previously (Eppig 1976).

Total RNA was extracted from cells using the ReliaPrep RNA Cell Miniprep System (PROMEGA, Madison, WI, USA), and reverse transcription was performed using the ReverTra Ace qPCR RT Master Mix with gDNA Remover kit (TOYOBO). Quantitative PCR was conducted using the THUNDERBIRD SYBR qPCR Mix (TOYOBO) and the StepOne Plus real‐time PCR system (Applied Biosystems, Foster City, CA, USA) in accordance with the manufacturer's instructions. Transcript levels were normalized to those of the reference gene *Rpl19* (ribosomal protein L19) using the 2^−ΔΔCt^ method (Livak and Schmittgen 2001). PCR was performed with an initial denaturation at 95°C for 10 min, followed by 40 cycles of denaturation at 95°C for 15 s and annealing/extension at 60°C for 1 min. Reactions were run in duplicate, and melting curve analysis was performed at the end of each reaction to avoid false‐positive signals. PCR products were additionally analyzed by agarose gel electrophoresis to confirm their sizes. The primers used in the present study are listed in Table 2. Amplification efficiency for each primer set was calculated from the slopes of the corresponding standard curves (Supporting Information S1: Figure S1).

**Table 2:** Primer sets used for qPCR.

Gene	Refseq Acc. no.	Forward primer sequence (5′−3′)	Reverse primer sequence (5′−3′)	Product size (bp)	Efficiency (%)
*Cyp19a1*	NM_007810	GGAGAACAATTCGCCCTTTCTT	GATGGACTCCACACAAACTTCCA	83	98.9
*Fshr*	NM_013523	CTCTCAGAATGATGTCTTGGAGGTAAT	GATGTACAGCAGATTGTTAGCCTTTTC	103	111.5
*Lhcgr*	NM_013582	GGATAGAAGCTAATGCCTTTGACAAC	TAAAAGCACCGGGTTCAATGTATAG	96	94.0
*Smpd2*	NM_009213	CTGGTGCTCCGTCTAAGTGG	GCATTCTTGGATGTGTGGTG	149	104.2
*Smpd4*	NM_001403422	AACGTTGCCAAGATTTGGTG	TAGGCATCGGAGATTCCAAC	134	94.1
*Rpl19*	NM_009078	CCGCTGCGGGAAAAAGAAG	CAGCCCATCCTTGATCAGCTT	103	109.2

### 2.8 In Vitro Growth of OGCs

The OGC culture medium was basic medium containing 5% FBS, 2% polyvinylpyrrolidone (PVP; Sigma‐Aldrich), 0.4826 mg/mL 2‐O‐(α‐d‐Glucopyranosyl)‐l‐ascorbic acid (Tokyo Chemical Industry Co. Ltd., Tokyo, Japan), and 5.0 ng/mL recombinant FSH (R&D Systems, Minneapolis, MN, USA).

OGCs were cultured in vitro as described previously (Akimoto et al. 2023). Briefly, OGCs of secondary follicles were isolated from the ovaries of 12‐day‐old mice using collagenase treatment (Eppig 1976). The OGCs were then placed on membrane inserts (Merck Millipore) and cultured in the OGC culture medium supplemented with 20 µM GW4869 or 0.2% DMSO. The cultures were maintained in a CO_2_ incubator at 37°C with 5% CO_2_ and 95% air for 9 days. The medium was changed every other day during the culture period.

### 2.9 In Vitro Maturation of Oocytes

To induce meiotic maturation, oocytes were cultured in basic medium supplemented with 5% FBS and 10 ng/mL recombinant epidermal growth factor (EGF; PeproTech, Cranbury, NJ, USA) (Akimoto et al. 2023). Maturation was evaluated by observing the extrusion of the first polar body under inverted microscopy after 16 h of culture.

### 2.10 sEV Treatment of MGCs

MGCs were collected as described above, seeded at 2 × 10^4^ cells per well in 96‐well plates, and incubated in basic culture medium supplemented with 3 mg/mL BSA, 5% FBS, and 10 nM milrinone, a phosphodiesterase inhibitor (Merck Millipore). After 24 h of culture, the medium was replaced with FBS‐free medium containing 100 ng/mL FSH, with or without 20 µM GW4869 and the MGC‐derived sEV fraction. Cells were then cultured for an additional 24 h.

In this experiment, the sEV fraction was isolated from conditioned medium pooled from two dishes (6 mL in total). For supplementation, the sEV fraction was resuspended in culture medium without any additional supplements (e.g., FSH, DMSO, or GW4869) and added at concentrations expressed as fold enrichment relative to the original conditioned medium volume. Specifically, a final resuspension volume of 6 mL corresponded to 1× enrichment, 600 µL to 10× enrichment, and 60 µL to 100× enrichment. In groups without sEVs, no resuspension medium was added.

The culture medium used in this experiment was the same as that used for EV isolation, except that it was supplemented with 0.2% DMSO, with or without FSH and/or GW4869. Total RNA extraction, cDNA synthesis, and quantitative PCR were performed as described above.

### 2.11 Statistical Analyses

Student's *t*‐test and the Tukey‒Kramer test were used for pairwise and multiple comparisons, respectively, using the JMP Pro Version 17 statistical analysis software (SAS Institute, Cary, NC, USA). Values of *p* < 0.05 were considered statistically significant. Values are presented as mean ± SEM.

## 3 Results

### 3.1 Effect of nSMase Inhibition on sEV Production by MGCs

**Figure 1:**
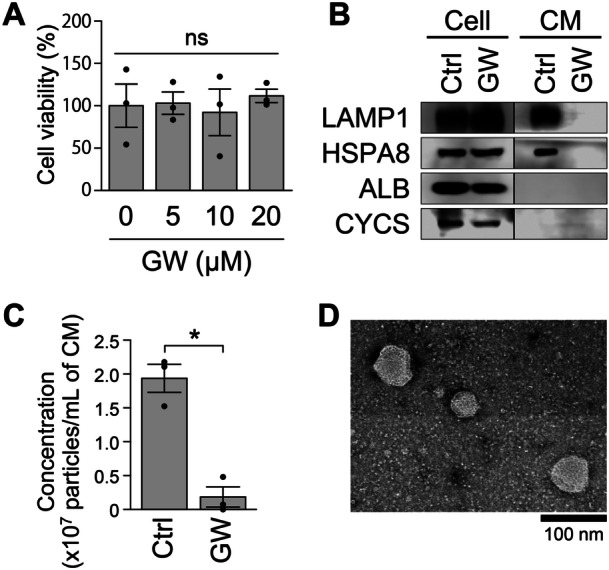
Effect of nSMase inhibition on sEV production by MGCs. (A) Effect of the nSMase inhibitor GW4869 (GW) on cell viability of MGCs (*n* = 3). All groups contained the same amount of solvent (0.2% DMSO) as the 20 µM GW4869 treatment. ns, not significant. (B) Western blot analysis for LAMP1, HSPA8, ALB, and CYCS. MGCs were cultured with GW4869 (20 µM) (GW) or its solvent DMSO (0.2%) (Ctrl) (*n* = 3). Cell, MGC lysate; CM, conditioned medium of MGCs. (C) NTA was performed to determine the particle number in the sEV‐enriched fraction isolated from the MGC‐conditioned medium. MGCs were cultured with GW4869 (20 µM) (GW) or its solvent (DMSO, 0.2%) (Ctrl). An asterisk denotes a significant difference (*p* < 0.05) (*n* = 3). (D) Representative TEM image of vesicles in the sEV‐enriched fraction, isolated from MGC‐conditioned medium, and observed using the negative‐staining method.

To investigate whether nSMase activity is required for sEV production by MGCs, MGCs were cultured with or without GW4869, and the presence of sEVs was assessed using Western blot analysis, NTA, and TEM (Figure 1). We selected a concentration of 20 μM GW4869 for the present study, as this dosage has been widely used in previous studies (Essandoh et al. 2015; Luberto et al. 2002). In addition, GW4869 treatment at concentrations up to 20 μM had no significant effect on the viability of MGCs (Figure 1A).

The proteins examined by Western blot analysis included LAMP1 (lysosomal‐associated membrane protein 1) and HSPA8 (heat shock protein 8, also known as HSC70) as positive markers and ALB (albumin) and CYCS (cytochrome c, somatic) as negative markers (Théry et al. 2018). As shown in Figure 1B, in the control group (0.2% DMSO), LAMP1 and HSPA8 were detected in both the sEV‐enriched fraction and MGC lysate, whereas ALB and CYCS were detected only in the MGC lysate. This confirmed that the isolated fraction contained sEVs and was free of contaminants such as apoptotic bodies and cell debris. Although 20 μM GW4869 supplementation did not affect the levels of LAMP1 and HSPA8 in MGCs, these proteins were barely detectable in the sEV‐enriched fraction from GW4869‐treated cultures than in the DMSO control. In addition, there were significantly fewer EV particles in the medium supplemented with GW4869 than in the DMSO control (Figure 1C). Moreover, TEM analysis revealed the presence of particles with diameters ranging from 50 to 150 nm in the sEV‐enriched fraction of the control group (Figure 1D).

These findings strongly suggest that sEV production by MGCs depends on nSMase activity.

### 3.2 Expression of Transcripts Encoding nSMase in Mouse Organs and During Granulosa Cell Development

**Figure 2:**
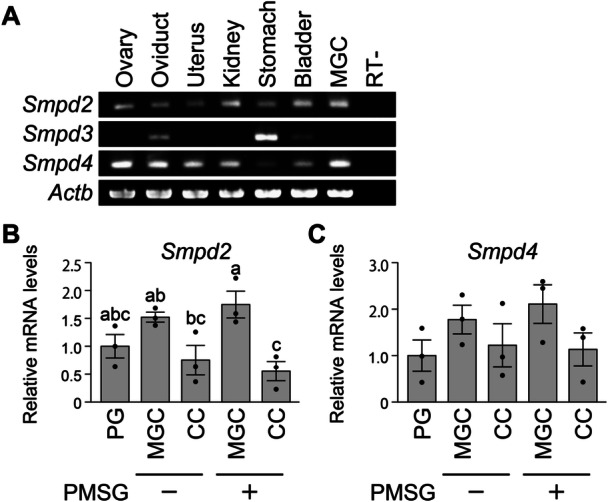
Expression of transcripts encoding nSMase in mouse organs and during granulosa cell development. (A) Expression of *Smpd2*, *Smpd3*, and *Smpd4* encoding nSMases was detected in various mouse organs by RT‐PCR (*n* = 3). The expression of *Smpd2* (B) and *Smpd4* (C) in preantral follicular granulosa cells (PG), cumulus cells (CC), and MGCs, with or without PMSG stimulation, was determined by qPCR (*n* = 3). Relative mRNA levels were calculated as the ratio of normalized mRNA (mRNA/*Rpl19*) in each sample to that in PG samples. Different letters (a, b, and c) denote significant differences (*p* < 0.05).

Three genes encode nSMase in mice: *Smpd2* (sphingomyelin phosphodiesterase, neutral 2), *Smpd3*, and *Smpd4*. To gain insight into which nSMase functions in MGCs, the expression of *Smpd2, Smpd3, and Smpd4* transcripts in mouse organs and MGCs was examined (Figure 2A). While *Smpd2* and *Smpd4* transcripts were detected in all organs examined, *Smpd3* expression was detected in only a few organs, including the oviduct, stomach, and bladder. Moreover, *Smpd2* and *Smpd4* transcripts, but not *Smpd3* transcripts, were detected in MGCs (Figure 2A).

Next, the kinetics of *Smpd2* and *Smpd4* expression during granulosa cell development were examined (Figure 2B,C). Granulosa cells were isolated from secondary follicles of 12‐day‐old mice and from small and large antral follicles of 3‐week‐old mice that were either unstimulated or stimulated with PMSG, respectively. The steady‐state levels of these transcripts were determined using qPCR. The levels of *Smpd2* transcripts tended to be higher in MGCs than in cumulus cells in small antral follicles (i.e., without PMSG stimulation) and were significantly higher in MGCs than in cumulus cells in large antral follicles (i.e., with PMSG stimulation) (Figure 2B). By contrast, *Smpd4* transcript levels exhibited a similar trend but did not reach statistical significance.

Therefore, *Smpd2* and *Smpd4*, but not *Smpd3*, are likely the major transcripts encoding nSMase in mouse MGCs.

### 3.3 Effects of Inhibiting nSMase Activity During OGC Development In Vitro

**Figure 3:**
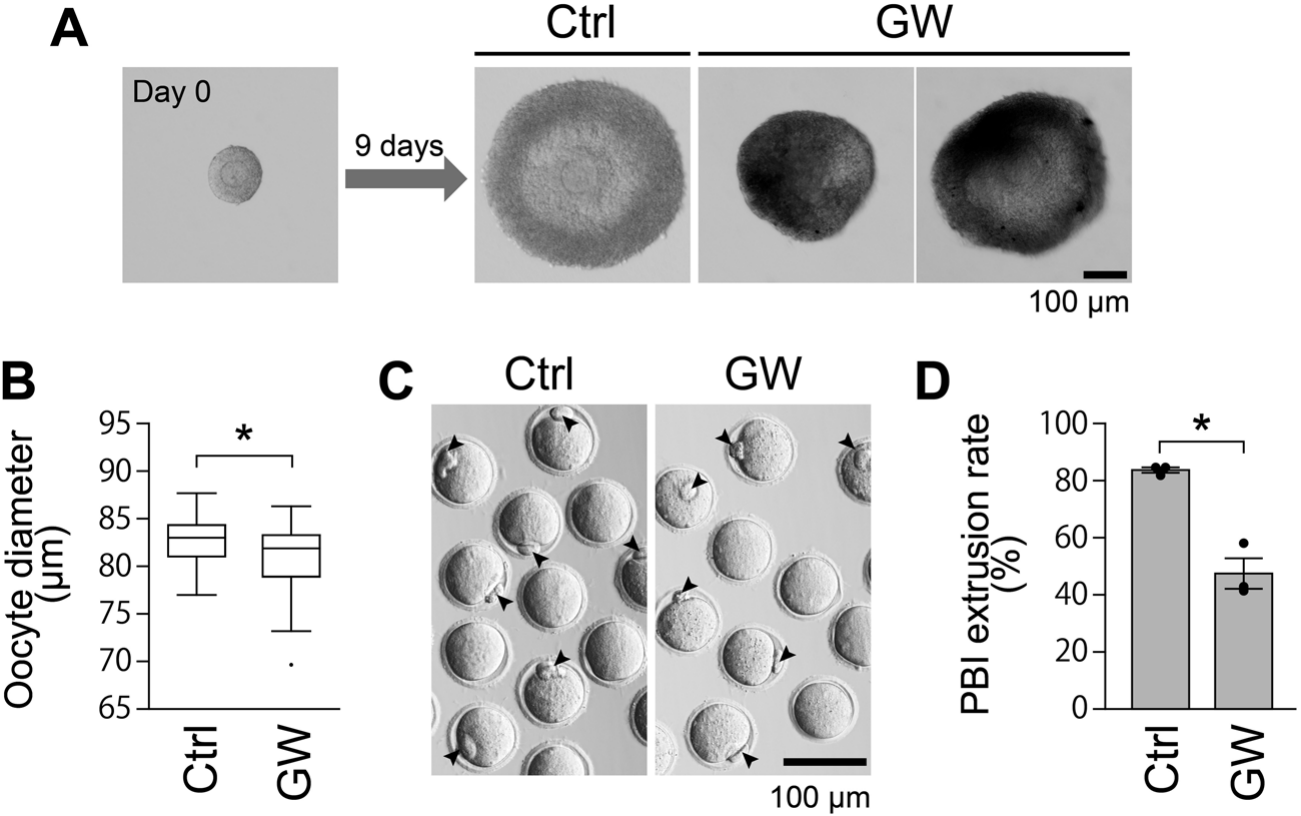
Effects of nSMase inhibition on the development of OGC in vitro. (A) Oocyte‒granulosa cell complexes (OGCs) derived from 12‐day‐old mice were cultured with the nSMase inhibitor GW4869 (20 µM) (GW) or its solvent DMSO (0.2%) (Ctrl) for 9 days. Representative photographs of OGCs before and after culture are shown. (B) The diameters of oocytes collected from OGCs cultured with or without GW4869 (20 µM) were measured. A total of more than 70 oocytes were evaluated in three independent experiments. An asterisk denotes a significant difference (*p* < 0.05). (C) Oocytes collected from OGCs grown with or without GW4869 (20 µM) were used for in vitro maturation (IVM) culture. Representative images of oocytes after IVM culture are shown. The arrowheads indicate the first polar bodies (PBI). (D) The rate of PBI extrusion was assessed. The data represent the average rate from three independent experiments in which a total of more than 70 oocytes were evaluated. An asterisk denotes a significant difference (*p* < 0.05).

To explore the role of sEVs in follicular development, we next examined how nSMase inhibition affects the in vitro growth of OGCs as a model (Figure 3). OGCs were isolated from secondary follicles of 12‐day‐old mice and cultured for 9 days in the presence or absence of GW4869. At the start of culture (Day 0), OGCs exhibited a simple structure, with the oocyte surrounded by a thin layer of granulosa cells. After 9 days of growth culture in the control group (containing 0.2% DMSO), OGCs developed an antral follicle‐like morphology, in which an antrum‐like cavity partitioned the granulosa cells into cumulus‐like and MGC‐like populations (Figure 3A). On the other hand, in the inhibitor‐treated group, the degree of morphological development varied among OGCs. Although some developed comparably to controls, the development of other OGCs was impaired. Overall, OGCs in the inhibitor‐treated group exhibited unclear antrum formation, and morphological differentiation between cumulus‐like and MGC‐like cells was poorly defined (Figure 3A).

We next assessed whether GW4869 supplementation during OGC culture affected oocyte growth and maturation. After 9 days, oocytes cultured with GW4869 had a slightly but significantly smaller mean diameter than controls (Figure 3B). Moreover, oocytes grown in the presence of GW4869 exhibited a significantly lower rate of first polar body extrusion than the controls (Figure 3C,D). Therefore, oocytes cultured in the presence of the inhibitor were smaller and exhibited reduced meiotic competence relative to the controls.

**Figure 4:**
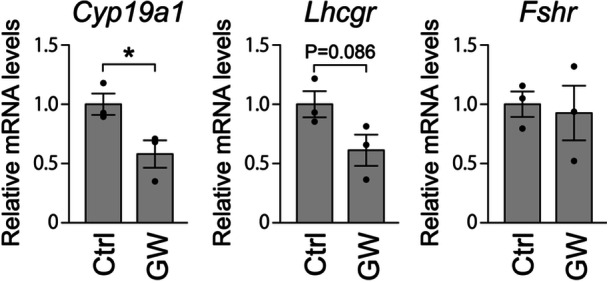
Effects of nSMase inhibition on gene expression in granulosa cells in vitro. MGCs were collected from OGCs after 9 days of culture, and *Cyp19a1*, *Lhcgr*, and *Fshr* levels were assessed by qPCR (*n* = 3). Relative mRNA levels were calculated as the ratio of *Rpl19*‐normalized mRNA levels in GW4869‐treated samples to those in control (Ctrl) samples. An asterisk denotes a significant difference (*p* < 0.05).

MGCs in large antral follicles typically express high levels of *Cyp19a1* (cytochrome P450, family 19, subfamily a, polypeptide 1), *Lhcgr* (luteinizing hormone/choriogonadotropin receptor), and *Fshr* (follicle‐stimulating hormone receptor) (Oktay et al. 1997; Peng et al. 1991; Stocco 2008), which serve as markers of MGC development. Accordingly, we compared the expression levels of these transcripts in MGCs developed with and without GW4869. The results illustrated that *Cyp19a1* transcript levels were significantly lower in MGCs cultured with the inhibitor than in the controls (Figure 4). *Lhcgr* expression also tended to be lower in the GW4869 group, whereas *Fshr* transcript levels were comparable between treated and control MGCs (Figure 4).

### 3.4 Effects of MGC‐Derived sEVs on In Vivo‐Grown MGCs

**Figure 5:**
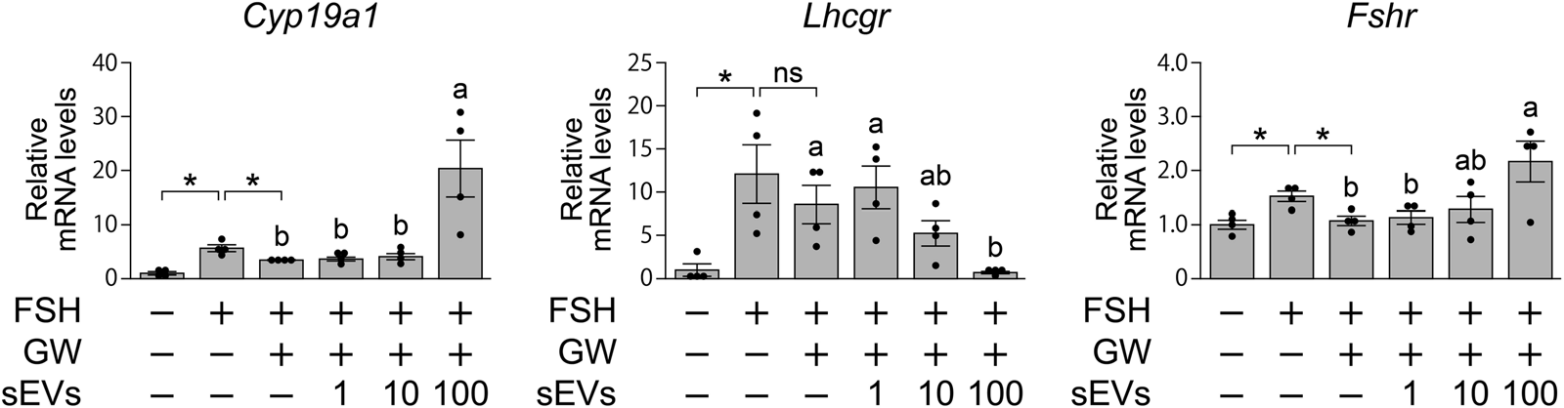
Effects of MGC‐derived sEVs on MGCs grown in vivo. In vivo‐developed MGCs, collected from PMSG‐stimulated ovaries, were cultured with GW4869 (20 µM) and MGC‐derived sEVs for 24 h, and *Cyp19a1*, *Lhcgr*, and *Fshr* levels were assessed by qPCR (*n* = 4). sEVs were added at equal, 10‐fold, and 100‐fold concentrations relative to the sEV concentration in medium conditioned by MGCs for 48 h. Relative mRNA levels were calculated as the ratio of normalized mRNA (mRNA/*Rpl19*) in each sample to that in untreated samples. An asterisk indicates a significant difference (Student's *t‐*test (*p* < 0.05). Different letters (a and b) denote significant differences according to the Tukey–Kramer test (*p* < 0.05). ns, not significant.

The above results suggest that MGC‐derived sEVs are necessary for full *Cyp19a1* expression in MGCs. To determine whether *Cyp19a1* expression could be restored by supplementing GW4869‐treated cultures with isolated sEVs, we attempted to add sEVs to the OGC culture. However, the quantity of sEVs necessary for sustained supplementation over the full 9‐day period was not attainable. We therefore assessed the effects of sEVs on monolayer cultures of MGCs isolated from in vivo‐developed follicles. Specifically, MGCs were isolated from the ovaries of PMSG‐injected mice and cultured in the presence of GW4869, with or without sEV‐enriched fractions prepared from untreated MGC‐conditioned medium. Transcript levels were measured 24 h after sEV addition. As shown in Figure 5, FSH stimulation significantly increased *Cyp19a1* expression, whereas GW4869 suppressed this induction. Supplementation with the sEV‐enriched fraction partially restored *Cyp19a1* expression in FSH‐ and GW4869‐treated MGCs, but only at a 100‐fold concentration relative to the original MGC‐conditioned medium. In contrast, *Lhcgr* expression was unaffected by GW4869 treatment but was significantly downregulated following sEV addition. Conversely, *Fshr* expression, which was reduced by GW4869, was significantly increased upon sEV treatment.

Although these findings suggest a role for MGC‐derived sEVs in promoting *Cyp19a1* expression, the results should be interpreted with caution (see Section 4 for details).

## 4 Discussion

Although sEVs in ovarian follicles appear to be produced primarily by MGCs, the pathway of sEV biogenesis within MGCs remains incompletely understood. In this study, we demonstrated that nSMase activity is essential for sEV production by MGCs in vitro and that *Smpd2* and *Smpd4* are the predominant transcripts encoding nSMase expressed by MGCs. Furthermore, sEVs produced by MGCs may contribute to the regulation of estrogen production by MGCs by promoting *Cyp19a1* expression within the follicle.

Our findings show that GW4869 treatment significantly reduced sEV production by MGCs to approximately one‐tenth of the control level, indicating that most sEVs in MGCs are generated through an nSMase activity‐dependent pathway. Although these findings do not necessarily exclude the involvement of the ESCRT pathway in sEV biogenesis by MGCs, they clearly demonstrate that inhibiting nSMase activity can effectively suppress sEV production within follicles. Although increasing attention has been paid to the role of sEVs in the ovary, most studies have relied on in vitro approaches, and in vivo analysis using genetic models remains limited. Our results support the feasibility of generating mouse models with reduced sEV production from granulosa cells by targeting *Smpd2*, *Smpd4*, or both genes. Systemic *Smpd4* knockout mice reportedly show reduced viability and male infertility (www.mousephenotype.org) (Groza et al. 2025), though female reproductive phenotypes have not been described. We are currently generating granulosa cell‒specific conditional *Smpd4* knockout mice. Analysis of these mice, along with *Smpd2/Smpd4* double knockout models, is expected to significantly advance our understanding of the physiological roles of sEVs within follicles.

While the current findings suggest that MGC‐derived sEVs may be necessary for the full expression of *Cyp19a1* by MGCs, these results should be interpreted with caution. Ceramide, synthesized via nSMase activity, plays diverse roles in maintaining normal ovarian function, including the regulation of apoptosis (Kaipia et al. 1996; Kim et al. 1999). Therefore, the observed reduction in *Cyp19a1* expression in MGCs following GW4869 treatment during OGC culture may not be attributable solely to suppressed sEV production but may also reflect other effects of ceramide synthesis inhibition. To address this, we investigated whether the reduction in *Cyp19a1* expression could be reversed by the addition of MGC‐derived sEVs. By using a monolayer culture of MGCs as a model, we confirmed that sEV supplementation rescued *Cyp19a1* expression. However, a measurable effect required sEVs at a concentration 100‐fold higher than that found in the original MGC‐conditioned medium. Substantial uncertainty remains regarding the efficiency of sEV recovery from culture supernatants, the functional integrity of isolated vesicles, and the differences in sEV concentrations between closely packed OGC cultures and monolayer cultures. Consequently, it is difficult to determine the physiological relevance of the sEV concentration used in this study. Nevertheless, the present findings, which demonstrate that suppression of sEV production reduces *Cyp19a1* expression in MGCs, whereas the addition of sEVs enhances such expression, strongly support the hypothesis that follicular sEVs regulate *Cyp19a1* expression in MGCs. In contrast, although sEVs altered *Lhcgr* and *Fshr* transcript levels in monolayer‐cultured MGCs, these effects were not observed in OGC cultures in which sEV secretion was suppressed. This discrepancy suggests that the observed effects of sEVs on the expression of these transcripts in MGC culture may not accurately reflect physiological mechanisms. A more rigorous assessment of sEV function will therefore require the combined application of nSMase inhibition and sEV‐mediated rescue experiments. Future studies employing the aforementioned genetic models should incorporate rescue experiments using OGC cultures or similar approaches, along with phenotypic analysis.

The present results show that GW4869 treatment during OGC development suppresses both oocyte growth and developmental competence. This suggests that sEVs are required for normal oocyte development, although other mechanisms, such as altered ceramide metabolism, may also be involved. Specifically, ceramide is hydrolyzed by ceramidases into sphingosine, which is subsequently phosphorylated by sphingosine kinase to form sphingosine‐1‐phosphate (S1P). It is well known that S1P promotes cell survival and proliferation, whereas ceramide is associated with cell death. Moreover, S1P has been implicated in multiple aspects of ovarian physiology, including follicular development, ovulation, and corpus luteum development (Hernández‐Coronado et al. 2019). Therefore, altered ceramide metabolism due to GW4869 treatment may be one of the reasons for the impaired oocyte development. Nonetheless, some evidence supports the idea that sEVs are vital for normal oocyte development. For example, while metabolic support, namely the provision of glycolysis products, from cumulus cells is mandatory for normal oocyte development (Emori and Sugiura 2014; Su et al. 2008; Sugiura et al. 2005), follicular fluid sEVs contain microRNAs and long noncoding RNAs implicated in glycolysis regulation (Cao et al. 2022; Zhou et al. 2023). In addition, while glycolysis in cumulus cells is regulated by factors secreted from oocytes (Sugiura et al. 2005, 2007), some unidentified factors, possibly sEVs, in follicular fluid are also required in pigs (Matsuno et al. 2016). Therefore, it is also possible that GW4869‐mediated sEV secretion inhibition disrupts the regulation of glycolysis in cumulus cells, thereby impairing oocyte development. Moreover, the present results suggest that sEVs are necessary for the expression of *Cyp19a1*, which encodes the enzyme responsible for estrogen production. Because estrogen is critical for cumulus cell development (Emori et al. 2013; Sugiura et al. 2010), it is possible that the observed attenuation of oocyte development was a consequence of reduced estrogen production due to impaired sEV‐mediated *Cyp19a1* expression. In this study, however, COCs from GW4869‐treated OGCs exhibited significantly weakened cumulus cell‒oocyte adhesion, precluding the collection of cumulus cells for gene expression analysis. Therefore, further investigation is required to disentangle the specific roles of sEVs and ceramide metabolism in oocyte development.

In summary, the present results show that nSMase activity, likely attributable to *Smpd2* and/or *Smpd4*, is primarily responsible for sEV biogenesis by MGCs, at least in vitro. This finding provides a foundation for future studies on sEV function in follicles using genetic models, although the results should be interpreted in light of potential effects of the ceramide synthesis pathway and related processes.

## Author Contributions

**Kodai Matsushita:** investigation, formal analysis, writing – original draft, writing – review and editing. **Yuta Matsuno:** investigation and formal analysis. **Kazuma Kita:** investigation and formal analysis. **Ayaka Ichikawa:** investigation and formal analysis. **Natsumi Maruyama:** investigation and formal analysis. **Wataru Fujii:** formal analysis and conceptualization. **Tsutomu Endo:** formal analysis and conceptualization. **Koji Sugiura:** formal analysis, conceptualization, writing – original draft, writing – review and editing.

## Ethics Statement

All experiments were approved by the Animal Care and Use Committee of the University of Tokyo.

## Conflicts of Interest

The authors declare no conflicts of interest.

## Supporting information

Supplementary Fig S1.

## Data Availability

The data relating to this article will be shared upon reasonable request to the corresponding author.
