# Supplementary material for: Impact of Neutral Sphingomyelinase Inhibition on Small Extracellular Vesicle Production by Mural Granulosa Cells and In Vitro Folliculogenesis in Mice
Source: Mol Reprod Dev. 2025 Oct 16;92(10):e70063. doi: 10.1002/mrd.70063 (PMC12529890; doi:10.1002/mrd.70063)
Supplement: Supplementary file 1 — Supplementary Fig S1. [file MRD-92-e70063-s001.docx]

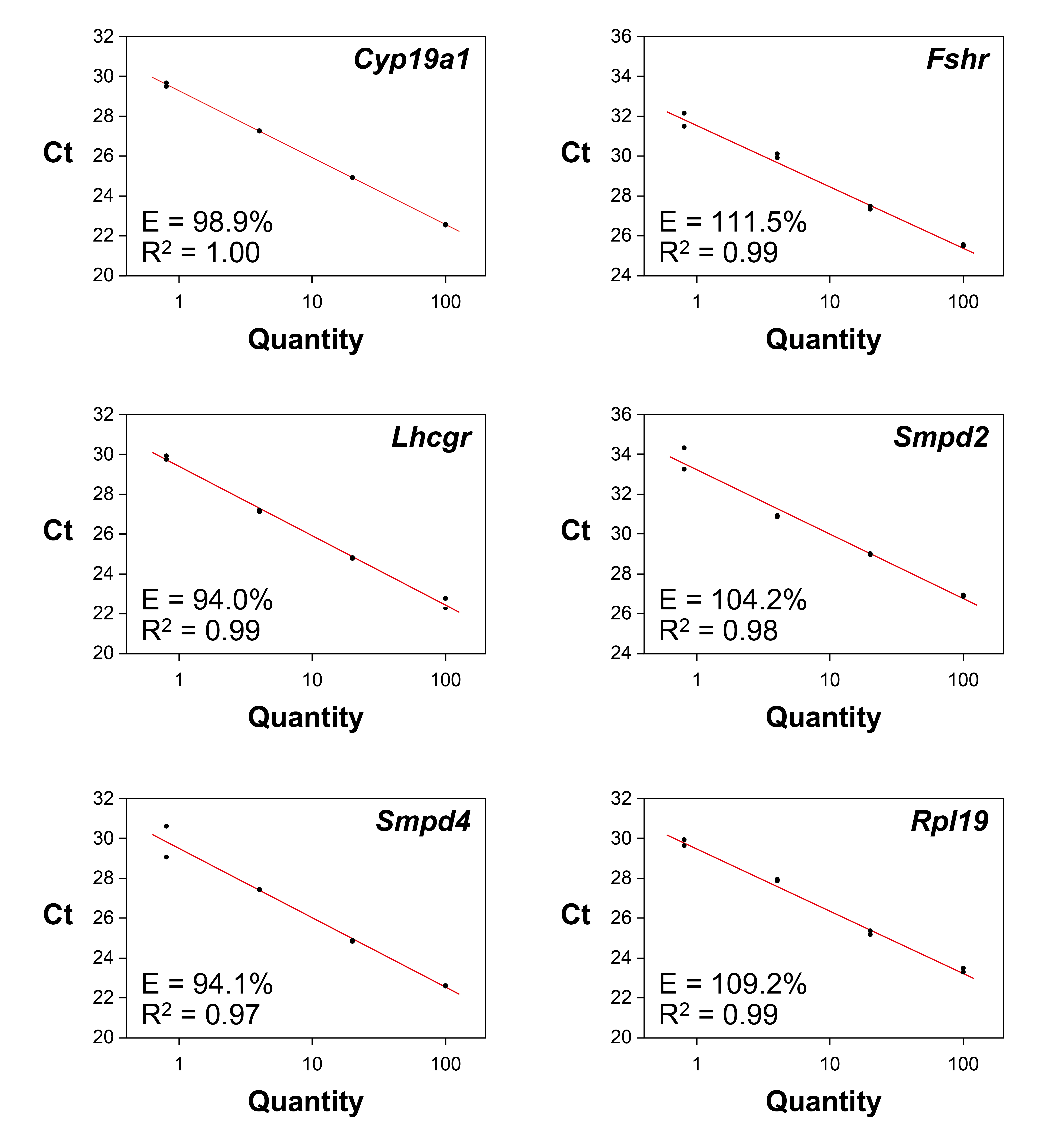


**Supplementary Figure S1. Standard curves for the qPCR primer sets.**

To evaluate the amplification efficiency of each primer set, a 5-fold serial dilution of cDNA derived from MGCs of PMSG-treated ovaries was prepared and used as the template for qPCR. Standard curves were generated by plotting the threshold cycle (Ct) values against the logarithm of the cDNA dilution. PCR efficiencies were calculated from the slope of the standard curve using StepOne Software (Applied Biosystems).
